# Polymorph Stability and Free Energy of Crystallization of Freely-Jointed Polymers of Hard Spheres

**DOI:** 10.3390/polym15061335

**Published:** 2023-03-07

**Authors:** Miguel Herranz, Javier Benito, Katerina Foteinopoulou, Nikos Ch. Karayiannis, Manuel Laso

**Affiliations:** Institute for Optoelectronic Systems and Microtechnology (ISOM) and Escuela Técnica Superior de Ingenieros Industriales (ETSII), Universidad Politécnica de Madrid (UPM), José Gutierrez Abascal 2, 28006 Madrid, Spain

**Keywords:** polymorphism, crystallization, hard sphere, Monte Carlo simulation, hexagonal close packed, face centered cubic, random walk, polymer, free energy, entropy

## Abstract

The free energy of crystallization of monomeric hard spheres as well as their thermodynamically stable polymorph have been known for several decades. In this work, we present semianalytical calculations of the free energy of crystallization of freely-jointed polymers of hard spheres as well as of the free energy difference between the hexagonal closed packed (HCP) and face-centered cubic (FCC) polymorphs. The phase transition (crystallization) is driven by an increase in translational entropy that is larger than the loss of conformational entropy of chains in the crystal with respect to chains in the initial amorphous phase. The conformational entropic advantage of the HCP polymer crystal over the FCC one is found to be $\Delta s_{ch}^{HCP-FCC}\approx 0.331\times 10^{-5}k$ per monomer (expressed in terms of Boltzmann’s constant *k*). This slight conformational entropic advantage of the HCP crystal of chains is by far insufficient to compensate for the larger translational entropic advantage of the FCC crystal, which is predicted to be the stable one. The calculated overall thermodynamic advantage of the FCC over the HCP polymorph is supported by a recent Monte Carlo (MC) simulation on a very large system of 54 chains of 1000 hard sphere monomers. Semianalytical calculations using results from this MC simulation yield in addition a value of the total crystallization entropy for linear, fully flexible, athermal polymers of Δs≈0.93k per monomer.

## 1. Introduction

Self-organization is a phenomenon of paramount importance in a plethora of systems related to physics, chemistry, biology, ecology, robotics, economy, cosmology and computer science [1,2,3,4,5,6,7,8,9,10,11]. In the most trivial example, self-organization manifests itself as the spontaneous, entropy-driven crystallization of hard bodies [12,13,14,15,16,17,18,19,20,21,22,23]. The formation of nematic liquid crystals, following Onsager’s theory [24,25], and the crystallization of hard objects [14,15,21,23,26,27,28,29,30,31], as first demonstrated by the pioneering simulations of Alder and Wainwright on spheres [32], are perhaps the two most prominent examples of entropy-driven transitions. Albeit the simplicity of the underlying physical model, numerous aspects of athermal crystallization remain still unclear, prominent among them being the final crystal polymorph as a result of the phase transition.

The structure of crystals of uniform, monomeric hard colloidal spheres, as investigated experimentally via light/X-ray scattering and confocal microscopy, is often found to be a random stacking of 2D hexagonal compact layers (rHCP) [12,33,34,35,36,37]. In some cases, depending strongly on experimental conditions, including factors like size polydispersity, shear, and gravity [34,35], quite perfect face-centered cubic (FCC) crystals are obtained [38], typically in samples grown over weeks or months, to allow for a slow annealing or aging for the transition rHCP→FCC to take place [12,13,39,40].

A widely accepted value of the entropy difference between the FCC and HCP polymorphs, supported by simulations [41,42,43,44,45], is $\approx 112\left(\pm 4\right)\times 10^{-5}k$ per particle, where *k* is Boltzmann’s constant. Variations from this value depend on the conditions under which the estimation is made, and especially on packing density (volume fraction) [41,42,44,46,47]. This very small value is qualitatively consistent with the experimentally observed sluggishness of the rHCP→FCC transformation. Accordingly, under a constant volume, most studies identify the rHCP polymorph as the final crystal structure [21,28,48,49] and perfection in the form of the FCC crystal is only rarely encountered [50,51]. Interestingly, neither perfect nor defective HCP crystals seem to have been observed in experiments and/or numerical simulations starting from densely packed amorphous samples.

The competition of the HCP and FCC polymorphs in monomeric packings of hard spheres has been extensively studied theoretically [41,42,43,44,45,47,52,53,54,55,56] and in computer simulations under a wide variety of conditions [53,57,58,59,60,61,62,63,64]. Much less is known about dense packings of hard-sphere polymers, both experimentally and theoretically. On the experimental side, while it is quite straightforward (e.g., through steric stabilization) to prepare systems that approach single hard-sphere behavior, the situation is significantly more complex for polymers of freely-jointed hard spheres, although promising experimental advances have been made especially with respect to granular [65,66], colloidal [67,68], and droplet [69] realizations. In parallel, the recent synthesis of giant polymer chains [70] allows their study at a significantly larger scale than the traditionally explored one. These experimental advances have been accompanied by computer simulations on linear chains made of hard spheres investigating, among other factors, the effect of packing density, chain length, chain stiffness, gaps in bond lengths and confinement [71,72,73,74,75,76,77,78,79,80,81].

Fully flexible polymers of hard spheres lacking any other type of interactions (i.e., without bending, torsional or bond length energetic contributions to the Hamiltonian) are athermal so that their equilibrium phase behavior is driven solely by entropy. Our previous work [75,76,77,78,79,80] showed that, rather counter-intuitively, starting from amorphous packings, freely-jointed chains of tangent hard spheres do indeed undergo spontaneous entropy-driven crystallization under a variety of conditions. In constant-volume simulations starting from an amorphous packing crystallization sets in when the increase in translational entropy of the monomers compensates for the loss of conformational chain entropy. The increase in monomer translational entropy is caused by an increase of the space available to monomers and of its isotropy [75,76], much as it happens in the transition from the isotropic fluid to the nematic liquid crystal mesophase in Onsager’s theory [24,25]. Due to the high complexity and computational demands of the algorithms needed to simulate long, athermal polymers at very high densities, the polymer crystals obtained in previous Monte Carlo (MC) simulations were made of short chains and tended to display a fivefold-free, but defect-ridden rHCP structure, so that it was not possible to establish which of the two competing crystal forms, HCP or FCC, was the thermodynamically stable one for chains of hard spheres.

Very recently we extended the studies by conducting unprecedentedly long MC simulations to study the entropy-driven crystallization of a large system of 54 chains of average length 1000 (in a number of spheres). In these isochoric simulations the initially amorphous configuration, after an early dominance of the HCP polymorph, passes to a transitory rHCP morphology and eventually reaches a stable FCC crystal of very high perfection [78]. In this work, which can be considered as a companion to [78], we present semi-analytical calculations of the free energy of crystallization of the stable FCC crystal and of its free energy advantage with respect to the HCP polymorph to support the observed simulation trends.

## 2. Methodology

### 2.1. Free Energy Difference between FCC and HCP Polymorphs

Athermal polymers are represented here as linear, freely-jointed chains of hard spheres of uniform size, σ, which is further the characteristic (unit) length of the system. According to the hard core model, spheres *i* and *j* interact with a pair-wise energy $u_{HS}\left(r_{ij}\right)$, given by:

(1) $$u_{HS}\left(r_{ij}\right)=\left\{\begin{array}{c} 0,r_{ij}\geq \sigma \\ \infty ,r_{ij}<\sigma \; \end{array}\right.$$

The spontaneous crystalization of polymers is conveniently studied in the (trivially) athermal version of the isochoric semigrand canonical ensemble $\left[VTN_{sites}\mu_{i}^{*}\right]$ in which total volume *V*, total number of sites $N_{sites}$ and a spectrum $\mu_{i}^{*}$ of chemical potentials are specified [82,83]. This ensemble naturally allows for polydispersity and for any desired distribution of polymer chain lengths spanning an arbitrary interval $l\in [l_{min},l_{max}]$, so that results are more generally valid than by assuming a specific distribution (Flory, uniform, etc).

We consider a system of *N* such polymer chains comprising a total number of monomers (also “sites”) $N_{sites}$. The chain length distribution is given by the number of chains $N_{l}$ of length $l\in [l_{min},l_{max}]$. In the following and for compactness of notation we also use $N_{l}$ to refer to the entire distribution. Although both $N_{sites}$ and *N* are fixed, the numbers $N_{l}$ of chains of each length *l* are fluctuating variables. The desired polymer length distribution results from imposing a suitable spectrum of chemical potentials, so that the constraints

(2) $$\begin{array}{c} N=\sum_{l=l_{min}}^{l_{max}}N_{l}\;\;\mathrm{and}\;\;N_{sites}=\sum_{l=l_{min}}^{l_{max}}lN_{l} \end{array}$$

hold, with *l* the counter over chain lengths. The partition function in the $\left[VTN_{sites}\mu_{i}^{*}\right]$ ensemble is [84]:

(3) $$\begin{array}{cc} & Y(V,T,N_{sites},\mu_{i}^{*})=\\ & ={\sum_{\mathrm{site}\;\mathrm{identities}}^{\infty }}^{\prime }\frac{q_{l_{min}}^{N}}{N!}\prod_{l=l_{min}}^{l_{max}}{\left(\frac{q_{l}}{q_{l_{min}}}\right)}^{N_{l}}\exp\left[\beta \mu_{l}^{*}N_{l}\right]V^{N}\\ & \times \frac{1}{N_{l_{min}}!\cdots N_{l_{max}}!}{\left(\frac{2\pi mkT}{h^{2}}\right)}^{3N_{sites}}\\ & \;\times Z(V,T,N_{l_{min}},\cdots ,N_{l_{max}}) \end{array}$$

where $q_{l}$ refers to the translational and internal contributions to the partition function for chain length *l*, and $Z\left(V,T,N_{l_{min}},\cdots ,N_{l_{max}}\right)=\int d^{3N}r\exp\left(-\beta U\left(r\right)\right)$ is the classical configurational integral. The athermal ensemble is trivially obtained by setting U(r)→∞ whenever at least one monomer overlap exists, and 0 otherwise (Equation (1)), so that the classical configurational integral reduces to a summation over equally probable microstates. The analytical evaluation of the general partition function (Equation (3)) is not feasible. It is however the starting point both for an analytical estimate of an upper bound of the entropy difference between crystal polymorphs, and for the development of MC algorithms.

Previous work [75,76] on the crystallization of chains of hard spheres has unequivocally established that the positions of chain monomers in the crystal fluctuate about the most probable sites of a well-defined polymorph (either HCP or FCC; other, non-compact crystal polymorphs do not appear in experiments or in simulations), while chains adopt random conformations compatible with the monomers fluctuating about these sites of the perfect crystal. Crystallization takes place because the loss of conformational entropy of the chains is more than compensated for by the increase in the positional/translational entropy of their monomers, even if these are forced to remain close to the sites of the crystal. The increase in monomer translational entropy is due to the larger and more isotropic volume translationally available to the monomers, in exact parallelism to what happens for monomeric hard spheres and in lyotropic phase transitions in liquid crystals as pioneered in [24] and further extended in [85,86,87].

If we ignore spatial fluctuations around their average positions, a crystal of hard sphere monomers consists of a single microstate (i.e., either the ideal FCC or HCP crystal, as specified by their lattices and bases). However, an interesting feature of the polymer crystal is that, again ignoring spatial monomer fluctuations, it possesses a large number of equally probable microstates: all possible multi-chain configurations in which the chains connect adjacent crystal sites without overlap and without leaving empty sites, while respecting monomer tangency along the chain backbone, are valid microstates. Thus, polymer crystal microstates are obtained to a very high degree of approximation as the product set of monomeric positional microstates (about the sites of the perfect crystal) and a highly multiply degenerate set of chain conformational microstates (all possible chain conformations that join the monomers in chains of the specified length).

The above description of the polymer crystal makes it possible to develop an accurate approximation to the partition function (Equation (3)) which is amenable to analytical calculations for the present case of crystals of freely jointed chains of hard spherical monomers by factorizing (Equation (3)) into a translational part for the individual monomers, as in a crystal of monomers, and a configurational part which accounts for chain connectivity and conformational variability. To this end we denote by $R_{i}^{X}$ the coordinates of the center of the *i*-th spherical monomer in a perfect crystal of monomers of polymorph *X* (where X= FCC or HCP) and by $R^{X}\equiv \left\{{⋃}_{i=1}^{N_{sites}}R_{i}^{X}\right\}$ the set of all monomer positions in the perfect crystal (i.e., $R^{X}$ is a list of $3N_{sites}$ Cartesian coordinates). The lowercase versions, namely $r_{i}^{X}$ and $r^{X}$, denote the coordinates of the *i*-th spherical monomer in a specific configuration of the real crystal (i.e., subjected to fluctuations), and the set of all $r_{i}^{X}$, respectively.

Given $R^{X}$ for a finite sample consisting of $N_{sites}$ of polymorph *X* there exists a finite set of polymer chain configurations that are obtained by tracing all sets of *N* nonoverlapping (i.e., simultaneously self-avoiding and mutually-avoiding) paths of the prescribed chain length distribution that connect all the $N_{sites}$ points of coordinates $R_{i}^{X}$. We now denote by $I_{jk}^{X}$ the *j*-th multichain configuration for the given $R^{X}$ and for the *k*-th chain size (discrete) distribution $N_{l}$, constrained by (Equation (2)). The integer counter *k* enumerates all possible chain distributions compatible with these constraints. Notice that it is not necessary to actually have the explicit complete finite set of possible chain distributions, nor its cardinality, because this set is independent of, and therefore the same for, all crystal polymorphs.

For any given, fixed numbering scheme of the $N_{sites}$ monomers and of the *N* chains, each $I_{jk}^{X}$ is (up to permutations of the site labels) a list of $N_{sites}+N-1$ integers which specify which and in which order crystal sites $R^{X}$ are occupied by monomers belonging to which chain ($N_{sites}$ integers), and which the chain lengths are (N−1 integers, because of the first constraint above). The union set $\xi_{k}^{X}\equiv \left\{{⋃}_{j}I_{jk}^{X}\right\}$ is then the set of all possible multichain configurations for a given chain length distribution, and the double union $\Xi^{X}\equiv \left\{{⋃}_{k}{⋃}_{j}I_{jk}^{X}\right\}$ is the complete, finite set of all possible multichain configurations for all possible chain length distributions compatible with $N_{l},l\in \left[l_{min},l_{max}\right]$, and constrained by (Equation (2)).

Accepting the separation of translational and conformational degrees of freedom in (Equation (3)) (an assumption whose plausibility will be quantitatively discussed below), and in terms of the previous definitions, the free energy/entropy difference $\Delta S^{X-Y}\equiv S^{X}-S^{Y}$ between polymorphs *X* and *Y* is, in units of *k*:

(4) $$\begin{array}{cc} & \Delta S^{X-Y}=\ln\frac{Z_{m}^{X}\left(V,r_{i}^{X}\right)}{Z_{m}^{Y}\left(V,r_{i}^{Y}\right)}\frac{\left|\Xi^{X}\right|}{\left|\Xi^{Y}\right|}=\\ & \ln\frac{Z_{m}^{X}\left(V,r_{i}^{X}\right)}{Z_{m}^{Y}\left(V,r_{i}^{Y}\right)}+\ln\frac{\left|\Xi^{X}\right|}{\left|\Xi^{Y}\right|}=\left(\Delta S_{m}^{X-Y}+\Delta S_{ch}^{X-Y}\right) \end{array}$$

where $Z_{m}^{X}\left(V,r_{i}^{X}\right)$ denotes the classical partition function for the monomeric crystal *X* and || denotes the cardinality of a set.

In physical terms, (Equation (4)) splits the evaluation of the entropy of the crystal of chains in two independent, additive contributions: the first one, $\Delta S_{m}^{X-Y}$ (corresponding to the first fraction in (Equation (4)) due to translational degrees of freedom of the monomers, as if they were not connected to form chains; the second one $\Delta S_{ch}^{X-Y}$ due to the number of ways the individual monomers can be connected into *N* chains of the specified length distribution under the condition that the monomers occupy all sites of the perfect crystal, i.e., the chains are both self-avoiding and mutually-avoiding, simultaneous random walks on the sites of polymorph *X*, so that no crystal sites are left unoccupied.

One advantage of (Equation (4)) is that $\Delta S_{m}^{X-Y}$ is the entropy difference between the FCC and HCP crystals of monomeric hard spheres, which is precisely known [41,43,44]. The problem is then reduced to the calculation of $\Delta S_{ch}^{X-Y}$, i.e., the chain configurational and conformational contribution to entropy. If $\Delta S_{ch}^{HCP-FCC}>\left|\Delta S_{m}^{FCC-HCP}\right|$ then the HCP crystal would be the stable polymorph, while FCC would be the thermodynamically preferred crystal form in the opposite situation.

Neither the cardinality $\left|\xi_{k}^{X}\right|$ nor consequently $\left|\Xi^{X}\right|$ are known for any crystal type; furthermore, their evaluation by exhaustive enumeration is conjectured to be an NP-complete problem [88] so that (Equation (4)) and $\Delta S^{FCC-HCP}$ cannot be evaluated exactly in the general case. It is however possible to establish an upper bound for $\Delta S_{ch}^{X-Y}$, which, if tight enough, would be sufficient to prove the stability of the FCC polymorph. An upper bound for $\left|\xi_{k}^{X}\right|$ is given by the product of the number of *N* neither self-avoiding nor mutually avoiding random walks of the given length distribution on the crystal sites of the polymorph, given by $\prod_{l_{min}}^{l_{max}}{\left(C^{X}\right)}^{N_{l}}$, with $C^{X}$ the coordination number of the crystal. However, since $C^{FCC}=C^{HCP}=12$ for both FCC and HCP, this bound predicts $\Delta S^{FCC-HCP}=0$ and does not allow to discriminate between the two crystal types.

A tighter and discriminating bound can be obtained by considering the finite set $c^{X}\left(l\right)$ of single self-avoiding random walks (SAWs) of length *l* on the sites of a given crystal *X*. The asymptotic dependence of $\left|c^{X}\left(l\right)\right|$ on *l* is of the exponential-power law type [89]:

(5) $$\begin{array}{c} \left|c^{X}\left(l\right)\right|=A\mu^{l}l^{\gamma -1} \end{array}$$

where *A* is the critical amplitude, μ the connective constant, and γ the critical exponent. For freely jointed chains, the asymptotic regime of (Equation (5)) is already attained for chains of very moderate length (l≈O(10)), so that (Equation (5)) is valid with excellent accuracy in the polymeric regime for which l≫O(10) ($N_{l}\approx O\left(100\right)-O\left(1000\right)$ in the present work).

Although it was conjectured that $\left|c^{HCP}\left(l\right)\right|=\left|c^{FCC}\left(l\right)\right|$, direct enumeration of SAWs demonstrates that this is exact only for l≤6 and first-order accurate for l>6; above that value of *l*, and hence in the polymeric regime of interest here, $\left|c^{HCP}\left(l\right)\right|>\left|c^{FCC}\left(l\right)\right|$ by a very small amount. We have calculated the cardinalities $\left|c^{FCC}\left(l\right)\right|$ and $\left|c^{HCP}\left(l\right)\right|$ as a function of SAW length *l* by exhaustive enumeration and also established their asymptotic behavior, which is accurately given by (5) with A=1.19, μ=10.07 and γ=1.134 for $\left|c^{FCC}\left(l\right)\right|$. For the HCP crystal, the values of these parameters are so similar to the FCC parameters, that it is numerically much preferable to express the minute difference between both in the expected decaying exponential form:

(6) $$\begin{array}{c} \left|c^{HCP}\left(l\right)\right|=\left|c^{FCC}\left(l\right)\right|\left[1+l\left(a_{1}-a_{2}\exp\left(-a_{3}l\right)\right)\right] \end{array}$$

with $a_{1}=3.31\times 10^{-6}$, $a_{2}=8.63\times 10^{-6}$, and $a_{3}=0.24$, for l>5 and $a_{1}=a_{2}=a_{3}=0$ for l≤5.

Since chains in the polymer crystal are random walks that are simultaneously mutually-avoiding and self-avoiding, and display ideal (non-SAW) statistics [90], their conformational ensemble is guaranteed to be a proper subset of the Cartesian product of the sets of all possible (self-avoiding and non-self-avoiding) individual chain conformations. Thus, for any given chain length distribution, $N_{l}$, the monotonicity of (Equation (5)) guarantees that the cardinality $\left|\xi_{k}^{X}\right|$ is strictly bounded from above by:

(7) $$\begin{array}{c} \left|\xi_{k}^{X}\right|<\prod_{l_{min}}^{l_{max}}{\left[c^{X}\left(l\right)\right]}^{N_{l}}<{\left[c^{X}\left(l_{max}\right)\right]}^{N} \end{array}$$

If we now denote by $N_{dist}$ the number of possible chain length distributions $N_{l}$ compatible with $N_{l},l\in \left[l_{min},l_{max}\right]$, and constrained by (Equation (2)), the following bound for $\left|\Xi^{X}\right|$ results:

(8) $$\begin{array}{c} \left|\Xi^{X}\right|<N_{dist}\sum_{k}{\left[c^{X}\left(l_{max}\right)\right]}^{N} \end{array}$$

where the sum is carried out over all possible chain length distributions $N_{l}$, with $l\in [l_{min},l_{max}]$, and constrained by (2). Note that $N_{dist}$ is independent, i.e., the same, for all crystal polymorphs and will cancel in any ratio of the type $\frac{\left|\Xi^{X}\right|}{\left|\Xi^{Y}\right|}$, such as Equation (9) below.

Because $\left|c^{HCP}\left(l\right)\right|$ is strictly larger than $\left|c^{FCC}\left(l\right)\right|$, because $\Xi^{HCP}\approx \Xi^{FCC}$ to first order, and because chain distributions are independent of polymorph type:

(9) $$\frac{\left|\Xi^{HCP}\right|}{\left|\Xi^{FCC}\right|}<\frac{\sum_{k}{\left[c^{HCP}\left(l_{max}\right)\right]}^{N}}{\sum_{k}{\left[c^{FCC}\left(l_{max}\right)\right]}^{N}}={\left(\frac{c^{HCP}\left(l_{max}\right)}{c^{FCC}\left(l_{max}\right)}\right)}^{N}$$

An upper bound for the entropy difference per monomer of the two polymorphs *X* and *Y*, $\Delta s_{ch}^{X-Y}$, is then given by:

(10) $$\begin{array}{cc} & \Delta s_{ch}^{HCP-FCC}=\ln\frac{\left|\Xi^{HCP}\right|}{\left|\Xi^{FCC}\right|}<\\ & <\frac{1}{\langle l\rangle }\ln\left[1+l_{max}\left(a_{1}-a_{2}\exp\left(-a_{3}l_{max}\right)\right)\right]\\ & =\left(a_{1}-a_{2}\exp\left(-a_{3}l_{max}\right)\right)<a_{1} \end{array}$$

where 〈l〉 is the number average chain length. The smallness of $a_{1}$ and $a_{2}$, and the polymeric regime (large *l*) allow us to only retain the leading term in the expansion of the logarithm, so that the last inequality follows from the asymptotic behavior of (Equation (6)). Then, to the second order:

(11) $$\Delta s_{ch}^{HCP-FCC}\approx 0.331\times 10^{-5}k$$

which implies that the chains in the HCP polymorph have higher conformational entropy than in the FCC crystal. However, this difference in conformational entropy is insufficient by more than two orders of magnitude to offset the translational entropic advantage $\approx 112\left(\pm 4\right)\times 10^{-5}k$ of the FCC polymorph with respect to the HCP crystal. Thus, the upper bound (Equation (9)), although conservative, is tight enough to unequivocally show that the FCC is the thermodynamically stable polymorph for crystals of linear, freely-jointed polymers of tangent hard spheres.

### 2.2. Monte Carlo Simulations

In order to provide computational support for the above results, based on semianalytical calculations, in a companion paper [78] we carried out unprecedentedly extensive MC calculations of very large systems deep in the polymeric regime (N=54 chains comprising $N_{sites}=54,000$ monomers, with a flat chain length distribution in the interval $\left[l_{min}=600,\;l_{max}=1400\right]$), at a volume fraction φ=0.56, and starting from a totally amorphous (random) packing. Observing spontaneous crystallization in a system of such size at that high volume fraction requires very efficient and proper configurational sampling, which is a very challenging task due both to the high density of the system and to the great length of the polymer chains.

One of the main motivations for simulating such a large system is to minimize potential finite size effects of small cells under periodic boundary conditions, which might conceivably lead to a crystallization advantage for the FCC polymorph due to the incommensurability of the cubic cell with the HCP crystal. Cell incommensurability is an irrelevant factor for such a large cubic cell, which is for all practical purposes just as compatible with the HCP crystals that appear in simulations as the cell usually known as “rhombic” (strictly speaking, hexagonal). Our simulation cell is significantly larger than individual chains and incipient HCP crystallites have ample possibility to freely nucleate and grow in the bulk of the system, irrespective of cell shape. For example, the size of the cubic cell is approximately 37 (measured in units of σ), more than twice the average radius of gyration $\langle R_{g}\rangle =16.5$, where 〈〉 denote the average over all chains and system configurations.

**Figure 1:**
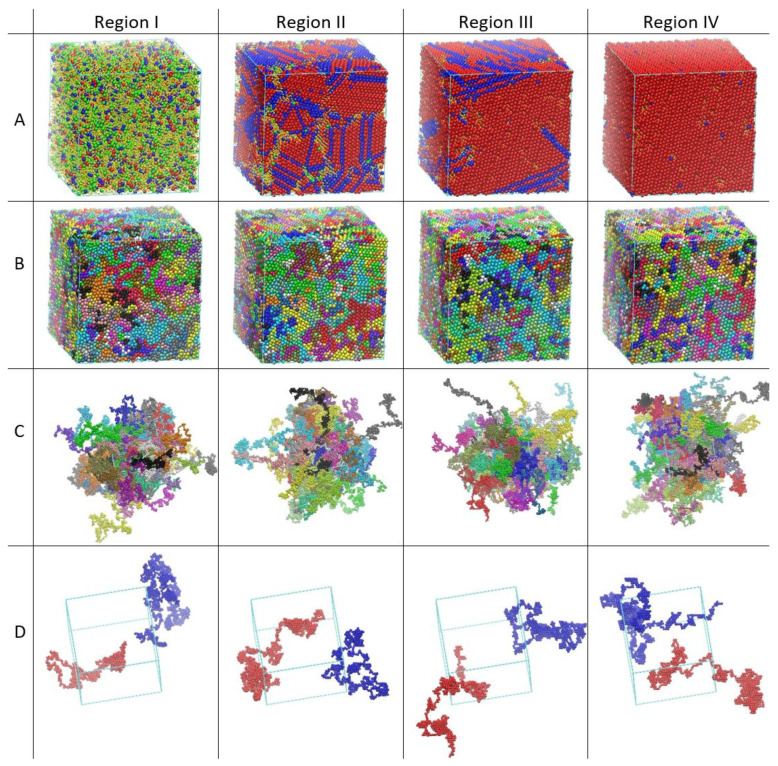
System snapshots along the MC simulation. From left to right: $1\times 10^{8}$ (Region I), $4\times 10^{11}$ (Region II), $8\times 10^{11}$ (Region III) and $1.4\times 10^{12}$ (Region IV, end of simulation) MC steps. From top to bottom: (**A**) Sites are colored according to their structural similarity as quantified through the CCE norm descriptor [93] with blue, red and green corresponding to sites with HCP, FCC and FIV character, respectively. Amorphous (AMO) sites are shown in yellow and with reduced dimensions for visual clarity. The stable FCC crystal (fourth, rightmost snapshot) is obtained in the steady state (up to fluctuations) MC production phase, after approximately $9\times 10^{12}$ MC steps; (**B**) Sites are colored according to their parent chain and are shown with wrapped coordinates, subjected to periodic boundary conditions; (**C**) Sites are colored according to their parent chain and are shown with coordinates fully unwrapped in space; (**D**) Two randomly selected chains are shown in red and blue with sphere coordinates fully unwrapped in space. Image panels created with the VMD software [95]. Details on the MC simulation and the corresponding trajectory can be found in [78].

In fact, as clearly seen in the following snapshots (Figure 1) HCP crystalline domains do actually form, and even temporarily surpass in abundance the FCC crystalline regions during part of the simulation. The disappearance of HCP domains and the final formation of a pure FCC crystal is not a consequence of using a cubic simulation cell. If anything, the cubic simulation cell would make a presumptively stable HCP crystal only very slightly more defect prone than a hexagonal one, but this effect would be completely obscured by the many imperfections that inevitably appear in all computer simulations of spontaneous crystallization. As a matter of fact, relatively perfect HCP crystals appear in simulations of crystallization in confined cubic cells where spatial restrictions appear in the form of flat parallel and impenetrable walls [79,80]. The cubic shape of the box can definitely be discarded as the cause of the final dominance and greater stability of the FCC polymorph.

System configurations are first generated and equilibrated through an MC scheme based on algorithms specially designed for the efficient sampling of polymer-based systems [83,91,92] and then characterized through the Characteristic Crystallographic Element (CCE) norm descriptor [93], both modules as implemented in the Simu-D software suite [94]. The MC trajectory is generated in the $\left[VTN_{sites}\mu^{*}\right]$ ensemble, where *V* is the volume of the cell, *T* is the temperature (inactive here due to the athermal nature of the model), and $\mu^{*}$ is the spectrum of chemical potentials used to control the distribution of chain lengths. The MC scheme consists of the following moves: (i) reptation (10%), (ii) rotation (10%), (iii) flip (34.8%), (iv) intermolecular reptation (25%), (v) configurational bias (20%), (vi) simplified end-bridging (0.1%) and (vii) simplified intermolecular end-bridging (0.1%), where numbers in parenthesis denote attempt percentages. Due to the very high volume fraction all local moves (i–v) are executed in a configurational bias pattern with the number of candidate configurations set equal to 50. Trial MC moves are accepted or rejected according to the modified Metropolis criteria as explained in Ref. [83]. The total duration of the MC simulation is $1.4\times 10^{12}$ steps with a record frequency of snapshots (frames) set at $1\times 10^{8}$ leading to a final trajectory being composed of 14,000 frames. The radial and orientational similarity of the local environment around each site with respect to reference crystals, as quantified through the CCE norm [93], classifies them as HCP, FCC, FIV (fivefold) or AMO (amorphous, or more precisely “not identified”) character. Throughout the present manuscript, blue, red, green and yellow will be used to represent the HCP, FCC, FIV and AMO sites and curves, respectively. The exact details on the methodology followed for the MC simulations and the successive structural analysis of the computer-generated system configurations can be found in the companion publication [78].

The evolution of the fraction of sites with HCP, FCC and FIV characters as a function of MC steps can be found in Figures 1 and 2 of [78]. Crystallinity is simply the summation of HCP and FCC fractions, while the degree of disorder of the system can be directly mapped into the fraction of AMO sites. Based on the observed trends the phase transition can be naturally split into 4 regions: (I) the rapid nucleation and growth of crystals with HCP and FCC character and the parallel decrease in the population of FIV and AMO sites, (II) the induction period where crystallization slows down, the population of HCP sites remains constant, the one of FCC increases very slowly while surviving FIV sites form characteristic linear assemblies corresponding to cyclic twin structures, (III) the FCC growth period which is accompanied by the elimination of sites with HCP similarity leading eventually to the formation of a single FCC crystal of very high perfection, (IV) the final, steady-state region, where within fluctuations, the established FCC crystal remains unaltered. The thresholds between the regions are marked by the significant slowdown in the crystal growth (I → II), the disappearance of fivefold sites (II → III), and the formation of the perfect FCC crystal (III → IV). A video showing the evolution of crystallization and the transition between the different crystal polymorphs can be found in the Supplementary Material, while system snapshots, corresponding to the four distinct regions, are presented in Figure 1. As can be seen in the supplementary video material and in the snapshots, starting from a purely amorphous, statistically homogeneous and isotropic configuration, the MC simulation is able to evolve the system through intermediate states of increasing crystallinity until a stable FCC polymorph of remarkable perfection is formed. In this final steady state the percentage of sites with FCC similarity exceeds 90% of the total population. A detailed explanation of the determination of the crystallographic type, of fivefold-symmetric, and of amorphous sites as implemented in the CCE norm descriptor is given in the companion paper [78] together with an analysis of the evolution including the entropic origins of crystal perfection. Based on the observed trends it is clear that driven by the minute FCC entropic advantage, the system shows a transition from the original disordered solid to the ordered crystal and spontaneously generates microstates of increasingly FCC character, until after approximately $9\times 10^{12}$ MC steps when all HCP crystalline sites disappear and crystallization reaches completion.

The initial predominance of the HCP polymorph (Regime I) and its eventual complete disappearance during the evolution towards a stable, perfect FCC polymer crystal is distinctly different from the evolution during crystallization of single spheres, where crystals of mixed FCC/HCP character are invariably obtained. This phenomenon is related to the different relative (meta)stability of intermediate states for crystals of single hard spheres, and of polymers of hard spheres, and is fully explained in the accompanying work [78].

## 3. Decorrelation of Translational and Conformational Degrees of Freedom

**Figure 2:**
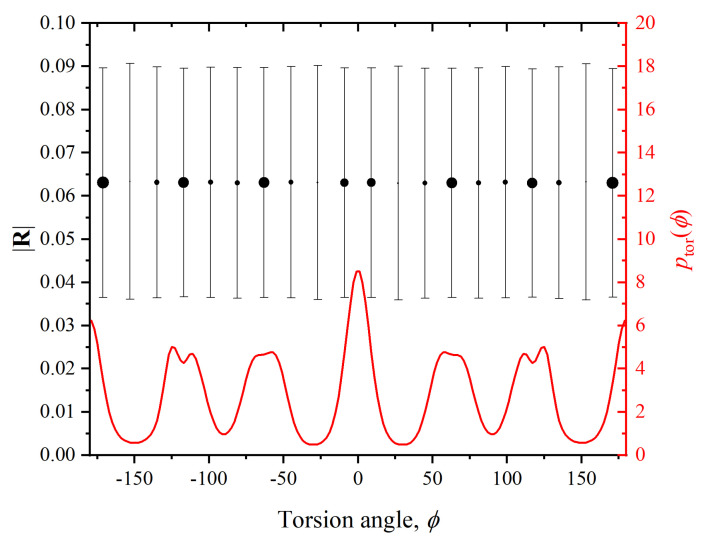
Left *y*-axis (black color): Distance |R| between a monomer and the centroid of its Voronoi cell, versus one of the torsion angles ϕ which belong to the same monomer, averaged over all frames in Region IV. Symbol size is proportional to the frequency of occurrence of the corresponding torsion angle ϕ. Right *y*-axis (red color): the probability distribution of torsion angles. Error bars are fluctuation amplitudes, angles have been grouped in 20 bins.

**Figure 3:**
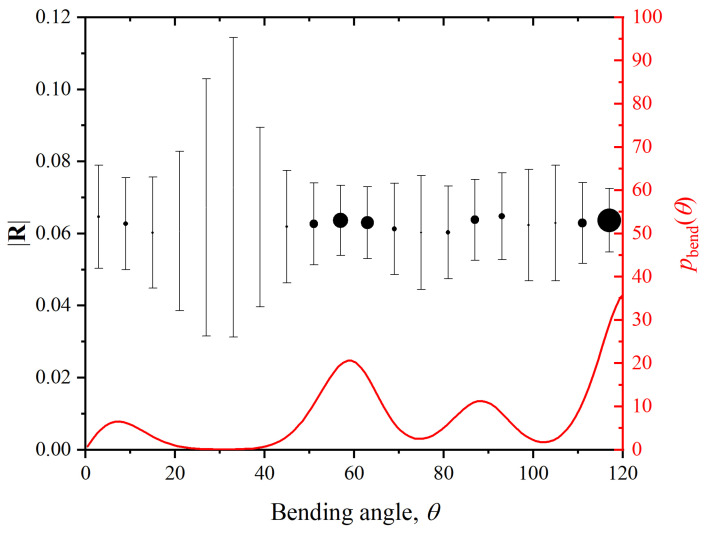
Left *y*-axis (black color): Distance |R| between a monomer and the centroid of its Voronoi cell, versus the bending angle θ of the monomer, averaged over Region IV. Symbol size is proportional to the frequency of occurrence of the corresponding bending angle θ. Right *y*-axis (red color): the probability distribution of bending angles. Error bars are fluctuation amplitudes, angles have been grouped in 20 bins.

We now examine the postulated decorrelation of translational and conformational (torsion and bending angles) degrees of freedom (d.o.f.’s), which is the basis of the calculations leading to the result of (Equation (11)). In order to test this hypothesis, we extract from the MC results the correlation between translational (monomer displacement) and conformational (bending and torsion angles of the chains) d.o.f.’s. To this end, we plot (Figure 2), for all monomers, and in Region IV the value of |R| against the torsion angles in which each monomer is involved, where |R| is the distance between a monomer and the centroid of its Voronoi cell:

(12) $$\begin{array}{c} \left|R\right|=\left(\frac{1}{N_{V}}\sum_{i=1}^{N_{V}}r_{i}\right)-r_{m} \end{array}$$

where $N_{V}$ is the number of vertices of the given Voronoi cell, $r_{i}$ are the position vectors of its $N_{V}$ vertices, and $r_{m}$ is the position vector of the monomer. Figure 3 is a similar plot of the value of |R| versus the bending angle whose vertex is located at the monomer.

In both figures the size of the symbols is proportional to the probability density of the particular value of the torsion (Figure 2) or bending (Figure 3) angle, and information on the fluctuation amplitude is included as error bars. For reference, the probability distributions of both ϕ and θ are included in the same plots. For both types of conformational degrees of freedom, the flatness of the curves attests to the independence of translational and torsional and bending conformational d.o.f.’s. For the torsional angle ϕ, the value of |R| is constant to better than 2%, and better than 5% for the bending angle. The values which deviate the most from the average (e.g., for $\theta =30^{o}$) correspond to angles that occur with extremely low frequency: $p_{bend}\left(\theta =30^{o}\right)\approx 0$, for which the symbol is invisibly small in Figure 3. Only very few instances of bending angles around this value exist, as the local chain geometry they produce is incompatible with the FCC cell, so they are very strongly suppressed in the crystal (flat portion around $\theta =30^{\circ }$ in Figure 3) and are for all practical purposes irrelevant.

From these two figures it can be concluded that the decorrelation between translational (|R| or its individual components, not shown) and conformational (ϕ, θ) d.o.f.’s is fulfilled to a sufficient degree to warrant their separation in the calculation of $\Delta s^{HCP-FCC}$, and the result (Equation (11)), between the two competing polymorphs HCP and FCC in the crystal.

In summary, although the free energy (entropy) advantage of the FCC over the HCP crystals of monomeric hard spheres is small ($\approx 112\left(\pm 4\right)\times 10^{-5}k$), the higher conformational entropy of chains in the HCP crystal with respect to FCC is even tinier $\approx 0.331\times 10^{-5}k$ and hence insufficient to make HCP the preferred crystal for chains. The results of the MC simulations strongly support the analytical result that the FCC polymorph is the thermodynamically stable phase for crystals of fully flexible chains of hard spheres.

## 4. Free Energy of Crystallization

Crystallization of fully flexible chains of hard spheres entails a reduction (relative to chains in an amorphous solid) in the number of conformations available to, and hence in the entropy of, the polymeric chains. However, just as in the case of monomeric hard spheres, the positional entropy of the individual monomers increases upon crystallization by a larger amount, so the overall result is still favorable for crystallization.

Thanks to the separation of translational and conformational d.o.f.’s, the increase in (monomeric) translational entropy upon crystallization of the chains can be taken as identical to the translational entropy increase of crystallization of monomeric hard spheres, which has been known for a long time: $\Delta s_{m}^{trans}=1.17k$ per monomer [32,96,97]. In order to compute the free energy (entropy) of crystallization of fully flexible polymers of hard spheres, it is now necessary to subtract from $\Delta s_{m}^{trans}$ the value of the conformational entropy lost by chains when they crystallize.

While an analytically exact calculation of the entropy of crystallization is not feasible, the results from the MC simulation suggest a feasible approximation method. First, the Kuhn length $b_{0}$ of the chains does not significantly change upon crystallization, as we have shown in [78]. The Kuhn length $b_{0}=1.52\pm 0.05$ turns out to be only 50% longer than the bond length, implying that the chains behave as identical freely jointed ideal (non-self-avoiding) chains when observed at length scales beyond a few bonds, in both the disordered solid and the ordered crystal.

**Figure 4:**
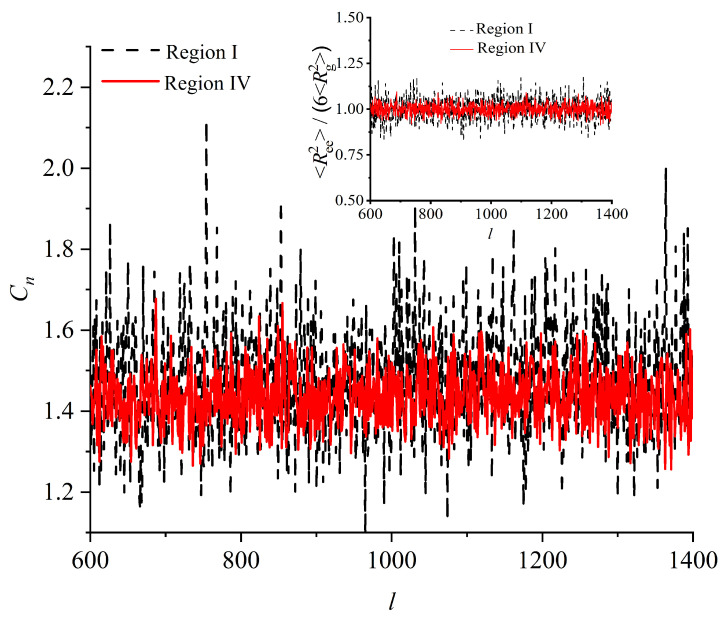
(Main panel) Characteristic ratio, $C_{n}$, and (inset) ratio of the mean square end-to-end distance divided by six times the mean square radius of gyration, $\frac{\langle R_{ee}^{2}\rangle }{6\langle R_{g}^{2}\rangle }$, as a function of chain length, *l*, in the disordered solid (Region I) and in the almost perfect FCC crystal (Region IV).

In Figure 4, we present the characteristic ratio $C_{n}$ (main panel), and the ratio $\frac{\langle R{ee}^{2}\rangle }{6\langle R_{g}^{2}\rangle }$ (inset), as a function of chain length, *l*. $\langle R_{ee}^{2}\rangle$ is the mean square end-to-end distance, and $\langle R_{g}^{2}\rangle$ is the mean square radius of gyration. Characteristic ratio is defined as $C_{n}=\frac{\langle R{ee}^{2}\rangle }{\left(l-1\right)\langle b_{len}\rangle }$, where $\langle b_{len}\rangle$ is the average bond length, as explained in Ref. [78]. Both are, within statistical uncertainty, independent of chain length $l\in [l_{min},l_{max}]$ and the same for both Regions, indicating that chains in the initial disordered solid (early Region I) and in the almost perfect FCC crystal (Region IV) are indistinguishable in the degree of coiling/flexibility and also equally ideal, since the ratio $\frac{\langle R_{ee}^{2}\rangle }{\langle R_{g}^{2}\rangle }$ adopts the value of 6 [98,99], as expected for ideal unperturbed polymers in the long-chain limit, as demonstrated originally by Debye [100] and then by Flory [90].

**Figure 5:**
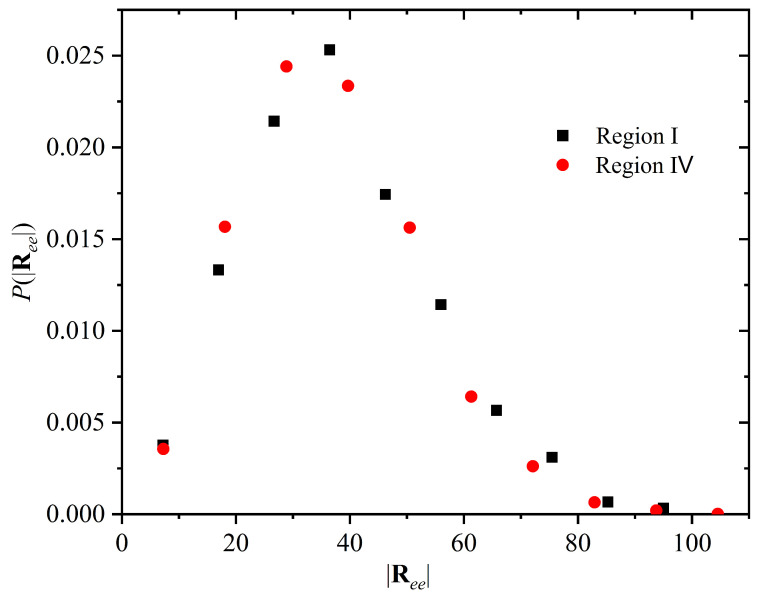
Probability distribution of the modulus of the end-to-end vector, $P(|R_{ee}|)$, for chains in the length interval l∈[970,1030] in the disordered solid (Region I) and in the almost perfect FCC crystal (Region IV). A small interval of *l* instead of single values of *l* has been used to obtain better statistics.

Furthermore, the distribution of the end-to-end vector of the chains, $P(|R_{ee}|)$ whose lengths lie within the small interval l∈[970,1030] (chosen as a representative, narrow interval of chain lengths) is also the same in the disordered solid and in the ordered crystal (Figure 5); the polymers neither shrink nor swell upon crystallization. These results unambiguously demonstrate that the large-scale features of the chains remain unchanged upon crystallization. In the two distinct phases (disordered solid and ordered crystal) chains differ only in their very small-scale features (a few bonds).

This statement may seem paradoxical at first sight but it is actually natural: the way chains crystallize (by having their monomers occupying on average the most probable sites of a crystal) uniformly (at all chain lengths) selects the chains that fulfill this condition from the other members of the ensemble. In slightly more precise terms, for a given crystal polymorph *X* and for a given chain length distribution, denoted by *k* (Section 2.1), the union set $\xi_{k}^{X}$ is (up to fluctuations) an equivalence class of the ensemble $\left[VTN_{sites}\mu_{i}^{*}\right]$ under the relation “monomer coordinates in a configuration of the ensemble $\left[VTN_{sites}\mu_{i}^{*}\right]$ coincide with site coordinates of crystal X”, a class for which any of the $I_{jk}^{X}$ can be taken as a representative. As a consequence of the underlying uniform (average) spatial density of crystal sites, the equivalence class retains all large-scale features of the full ensemble.

Figure 4 and Figure 5 clearly demonstrate the similarity of chains in the disordered solid and in the almost perfect FCC crystal; to convey an intuitive impression for this similarity we show in Figure 1 snapshots of the whole system with chains being subjected to periodic boundary conditions (second row), being fully unwrapped in space (third row) for all four Regions exhibited during crystallization. In addition, only two, randomly selected, chains are visualized for each system for clarity in the bottom (fourth row) of Figure 1. In all cases, the chain features, down to a few bond lengths, are indistinguishable so that it is impossible to tell apart polymers in the original amorphous solid and in the various crystal polymorphs including the perfect FCC crystal.

Since conformational differences between chains in the disordered solid and the FCC crystal are overwhelmingly local, a calculation of the loss of conformational entropy upon crystallization $\Delta s_{m}^{conf}$ can be made, inspired by Flory’s Rotational Isomeric State (RIS) theory [90] by considering continuously varying bending and torsional angles. Although the absolute value of the conformational entropy cannot be computed exactly either in the amorphous or in the crystal phases, the previous arguments make it possible to estimate the difference in conformational entropy between the amorphous solid and the crystal by considering up to next-next-next-nearest neighbors, i.e., a four-monomer portion of chain, beyond which chains remain essentially unaltered upon crystallization.

**Figure 6:**
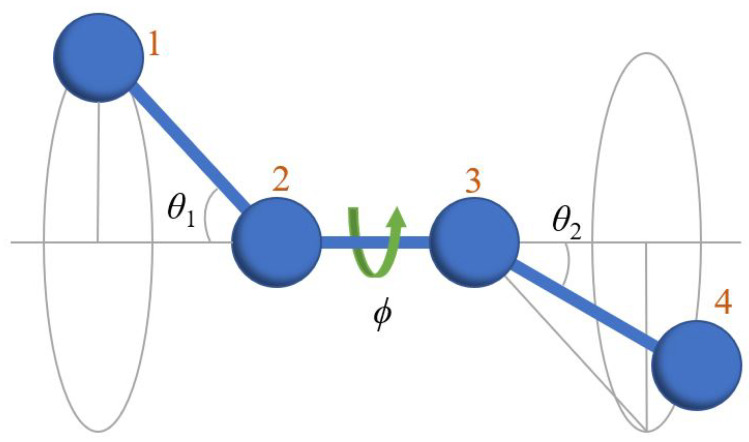
Definition of bending angles $\theta_{1},\theta_{2}$ and torsion angle ϕ. These angles are also used in the joint orientational functions of bending and torsion angles $f_{bt}\left(\theta_{1},\phi ,\theta_{2}\right)$ (see Section 4). The angle ϕ gives the rotation around the line defined by monomers 2-3 and is measured with respect to the plane defined by the three successive bonds 1-2-3.

We denote by $f_{bt}^{am}\left(\theta_{1},\phi ,\theta_{2}\right)$ and $f_{bt}^{cr}\left(\theta_{1},\phi ,\theta_{2}\right)$ the joint orientational distribution functions of bending and torsion angles for a four-monomer chain segment (see Figure 6) in the amorphous solid and in the crystal, respectively. The angles $\theta_{1},\theta_{2}$ are two consecutive bending angles defined by monomers 1-2-3, and 2-3-4 (see Figure 6), and ϕ is the torsional angle defined by monomers 1-2-3-4.

The configurational entropy difference upon crystallization (in units of Boltzmann’s constant *k*) can be expressed in terms of the integrals of the joint orientational functions [101] by:

(13) $$\begin{array}{c} \Delta s^{conf}=\int du^{\left(1\right)}\int du^{\left(2\right)}\int_{0}^{2\pi }d\phi f_{bt}^{cr}\left(\theta_{1},\phi ,\theta_{2}\right)\ln f_{bt}^{cr}\left(\theta_{1},\phi ,\theta_{2}\right)-\\ \int du^{\left(1\right)}\int du^{\left(2\right)}\int_{0}^{2\pi }d\phi f_{bt}^{am}\left(\theta_{1},\phi ,\theta_{2}\right)\ln f_{bt}^{am}\left(\theta_{1},\phi ,\theta_{2}\right) \end{array}$$

where the integral operation ∫⋯du is equivalent to ∫∫⋯sinθdθdϕ with integration ranges 0≤θ≤π and 0≤ϕ<2π. The integration over the unit vectors $u^{\left(1\right)},u^{\left(2\right)}$ and the torsion angle ϕ are tantamount to carrying out the integration over all possible states of the four-monomer segment.

The orientational functions $f_{bt}^{cr}\left(\theta_{1},\phi ,\theta_{2}\right)$ and $f_{bt}^{am}\left(\theta_{1},\phi ,\theta_{2}\right)$ are unknown a priori but can be evaluated numerically as averages over Region IV (final FCC crystal) and over the initial amorphous state of Region I, respectively, and discretized on regular integration meshes of increasing resolution ranging from 20×20×20 up to 60×60×60 for all $\theta_{1},\phi ,\theta_{2}$ in order to ensure numerical convergence of the integrals.

**Figure 7:**
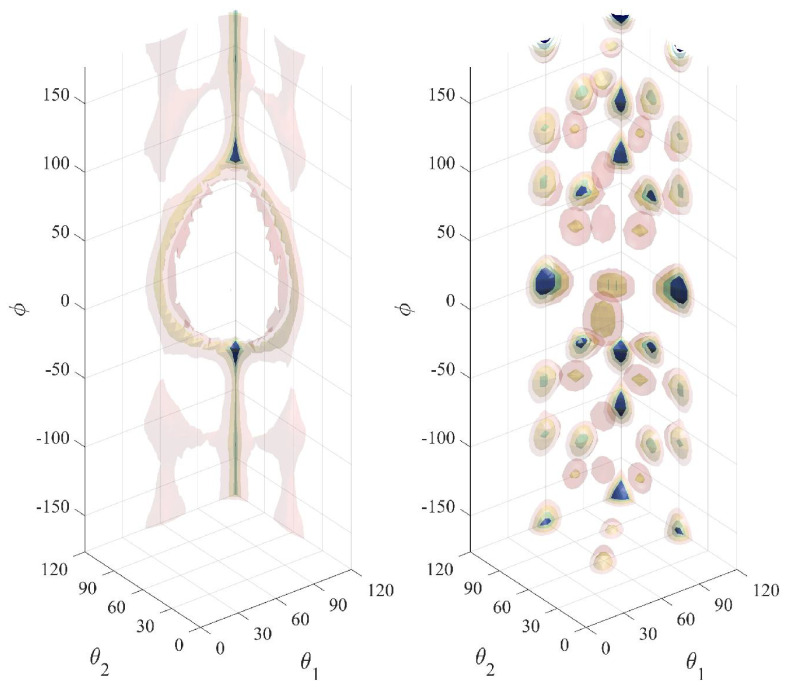
Isosurface representation of the integrands in Equation (13) in the amorphous $f_{bt}^{am}\left(\theta_{1},\phi ,\theta_{2}\right)$ (left), and in the crystal $f_{bt}^{cr}\left(\theta_{1},\phi ,\theta_{2}\right)$ (right). Isosurface coloring corresponds to $1.5\times 10^{-5}$ (transparent pink), $3.0\times 10^{-5}$ (transparent yellow), $6.0\times 10^{-5}$ (transparent green), $9.0^{-5}$ (solid blue).

**Figure 8:**
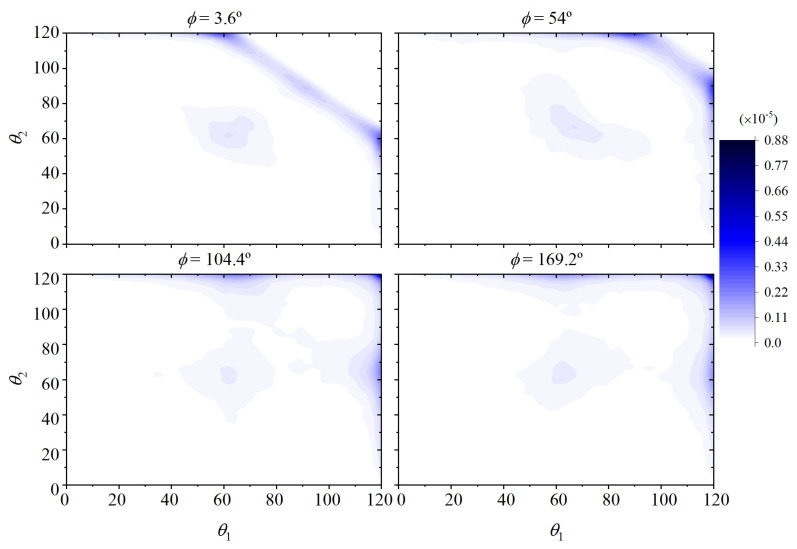
Sections of the joint orientational functions of bending and torsional angles in the initial amorphous state $f_{bt}^{am}\left(\theta_{1},\phi ,\theta_{2}\right)$ for four values of the torsion angle ϕ.

**Figure 9:**
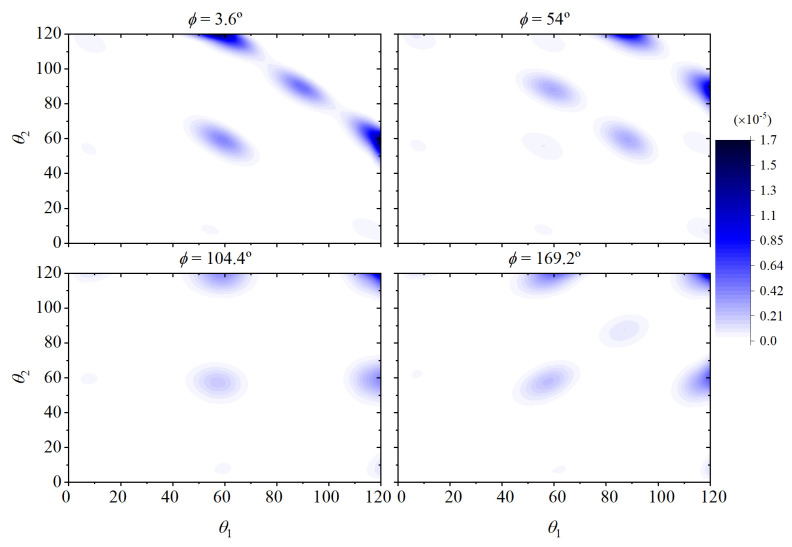
Sections of the joint orientational functions of bending and torsional angles in the stable FCC polymorph $f_{bt}^{cr}\left(\theta_{1},\phi ,\theta_{2}\right)$ for four values of the torsion angle ϕ.

Figure 7 (as 3D isosurfaces), Figure 8 and Figure 9 (as sections of these isosurfaces at several values of ϕ) illustrate how the preferred bending and torsional angles differ between the amorphous and the crystalline states. As can be seen in Figure 7, specific triads of $\theta_{1},\phi ,\theta_{2}$ appear in the stable crystalline polymorph with significant frequency. The highly probable combinations of $\theta_{1},\phi ,\theta_{2}$ (disconnected, high probability regions in the right panel of Figure 7) are responsible for the instantaneous positions of the monomers fluctuating about the sites of the ideal FCC crystal.

A numerical evaluation of Equation (13) yields an entropy loss (i.e., the entropy of the chains in the crystal state is lowered by the loss of conformational freedom) $\Delta s^{conf}=-0.24k\pm 0.04$ per monomer. This figure is significantly lower (in absolute value) than the translational entropy increase due to the formation of the crystal, which is $\Delta s_{m}^{trans}=1.17k$ per monomer, as mentioned above. The free energy of crystallization of fully flexible chains of hard spheres (measured by the net increase in entropy per monomer upon crystallization of the athermal system) is then $\Delta s_{m}=\Delta s_{m}^{trans}+\Delta s^{conf}\approx 0.93k$, which is the result we sought. This value is still more than sufficient to drive the phase transition, as in monomeric hard spheres, in spite of the loss of chain conformational entropy.

## 5. Conclusions

We have presented semianalytical calculations of the free energy of crystallization of linear, freely jointed chains of tangent hard spheres, as well as of the free energy difference between the FCC and HCP polymorphs. The calculations are based on a separation of degrees of freedom, i.e., a decoupling of chain conformational and monomer translational degrees of freedom. This postulate is confirmed to hold within narrow error margins. The calculations predict a small advantage of the FCC crystal over the HCP, which makes FCC the thermodynamically stable one. This prediction is consistent with the results of very long MC simulations on a large simulation cell comprising 54,000 monomeric sites assembled in 54 chains, as presented in the companion publication [78].

In addition, chain conformations in the initial amorphous and the final crystal phases are found to be identical in all large-scale features and differ only locally, at quite small length scales of very few bonds. This fact allows a calculation of the loss of conformational entropy per monomer upon crystallization, $\Delta s^{conf}=-0.24k\pm 0.04$. This decrease is less than the increase in translational entropy of the monomers, ($\Delta s_{m}^{trans}=1.17k$ per monomer), so crystallization still increases the overall free energy (entropy) in agreement with the observed spontaneous formation of crystals of polymeric chains. Thus, the calculated entropy of crystallization of freely jointed chains of hard spheres turns out to be $\Delta s_{m}\approx 0.93k$.

## Data Availability

The data presented in this study are openly available in Zenodo http://zenodo.org/record/7682587#.Y_z9zh_MIR8 (Last accessed: 3 March 2023).

## Supplementary Materials

The following supporting information can be downloaded at: https://www.mdpi.com/article/10.3390/polym15061335/s1, Video S1: N1000 crystallization.
